# Risk prediction of stroke-associated pneumonia in acute ischemic stroke with atrial fibrillation using machine learning models

**DOI:** 10.3389/frai.2025.1595101

**Published:** 2025-05-20

**Authors:** Tai Su, Peng Zhang, Bingyin Zhang, Zihao Liu, Zexing Xie, Xiaomei Li, Jixiang Ma, Tao Xin

**Affiliations:** ^1^School of Public Health and Management, Shandong First Medical University & Shandong Academy of Medical Sciences, Jinan, China; ^2^Department of Neurology, The First Affiliated Hospital of Shandong First Medical University, Jinan, China; ^3^Shandong Institute of Neuroimmunology, Jinan, China; ^4^Shandong Provincial Center for Disease Control and Prevention, Jinan, China; ^5^Department of Neurosurgery, Shandong Provincial Hospital, Shandong First Medical University, Jinan, China; ^6^Department of Neurosurgery, The First Affiliated Hospital of Shandong First Medical University, Jinan, China

**Keywords:** acute ischemic stroke, atrial fibrillation, stroke-associated pneumonia, machine learning model, nomogram

## Abstract

Stroke-associated pneumonia (SAP) is a serious complication of acute ischemic stroke (AIS), significantly affecting patient prognosis and increasing healthcare burden. AIS patients are often accompanied by basic diseases, and atrial fibrillation (AF) is one of the common basic diseases. Despite the high prevalence of AF in AIS patients, few studies have specifically addressed SAP prediction in this comorbid population. We aimed to analyze the factors influencing the occurrence of SAP in patients with AIS and AF and to assess the risk of SAP development through an optimal predictive model. We performed a case-control study. This study included 4,496 hospitalized patients with AIS and AF in China between January 2020 and September 2023. The primary outcome was SAP during hospitalization. Univariate analysis and LASSO regression analysis methods were used to screen predictors. The patients with AIS and AF were randomly divided into a training set, validation set, and test set. Then, we established logistic regression (LR), random forest (RF), support vector machine (SVM), and extreme gradient boosting (XGBoost) models. The accuracy, sensitivity, specificity, area under the curve, Youden index and *F*_1_ score were adopted to evaluate the predictive value of each model. The optimal prediction model was visualized using a nomogram. In this study, SAP was identified in 10.16% of cases. The variables screened by univariate analysis and LASSO regression, variables such as coronary artery disease, hypertension, and dysphagia, identified by univariate and LASSO regression analyses (*p* < 0.05), were included in the LR, RF, and SVM. The LR model outperformed other models, achieving an AUC of 0.866, accuracy of 90.13%, sensitivity of 79.49%, specificity of 86.11%, *F*_1_ score of 0.80. A nomogram based on the LR model was developed to predict SAP risk, providing a practical tool for early identification of high-risk patients, and enabling targeted interventions to reduce SAP incidence and improve outcomes.

## Introduction

Stroke is the second leading cause of death worldwide and the primary cause of disability-adjusted life years (DALYs) in China (GBD 2019 Stroke Collaborators, 2021; Zhao et al., 2023). AIS is a critical nervous system disorder caused by thrombosis and embolism that block cerebral arteries, accounting for 85% of stroke types (Cerami and Perani, 2015). AIS is associated with the development of neuroinflammation and may also arise from cerebral hemodynamic abnormalities. AF is a significant contributor to the development of stroke (Kelley and Kelley, 2021). Approximately 24% of patients with AIS also have AF, and individuals with AF face a risk of AIS that is 4–5 times higher than those without AF which increases the overall risk of AIS for these patients (Kimura et al., 2018). Additionally, the inflammatory response plays a crucial role in both AIS and AF, as well as their related complications (Xu et al., 2024).

SAP refers to pneumonia occurring within 7 days of admission in non-ventilated stroke patients (Ji et al., 2013). It is a common and serious complication in stroke patients, significantly increasing morbidity and mortality. The global incidence rate of SAP ranges from 7 to 38%, with the acute mortality rate reaching as high as 30–40% (Gittins et al., 2023; Teh et al., 2018; Yu et al., 2016; Bai et al., 2020; Yu et al., 2016). This significantly impacts patients’ prognosis, prolongs hospitalization time, and increases the medical burden (Koennecke et al., 2011). Studies have shown that the frequency of SAP is significantly higher in patients with AF compared to non-AF patients (9.8% vs. 5.3%), which may be related to systemic inflammatory responses and immune dysfunction associated with AF (Keller et al., 2020).

As a core technology of artificial intelligence, machine learning (ML) refers to the ability to recognize patterns and learn from data (Ting Sim et al., 2023). In recent years, it has been widely applied in various areas, including disease prediction, disease prognosis assessment, disease-assisted diagnosis, and health management (Shurrab et al., 2024). ML models, including extreme gradient boosting (XGBoost), support vector machines (SVM), random forest (RF), logistic regression (LR), and deep neural networks (DNN), can capture complex non-linear relationships and identify unknown correlations in big data, providing deeper insights. RF is a non-parametric method based on the bagging principle, which adopts the outputs of integrating algorithms of multiple decision trees. This approach enhances the overall model performance by strengthening multiple weak classifiers, making it more effective than a single decision tree (Hu and Szymczak, 2023). SVM introduces the concept of kernel functions and employs the principle of structural risk minimization. This allows for nonlinear decision-making in the original space by identifying a linear hyperplane within a high-dimensional space (Si et al., 2023). The XGBoost algorithm is a large-scale machine-learning algorithm that represents an efficient and extensible variant of gradient enhancement (Li et al., 2022).

The purpose of this study is to compare the predictive performance of various ML models for SAP in patients with AIS and AF by using predictive factors effectively. This approach aims to facilitate the early identification of high-risk groups in clinical settings, support individualized treatment, improve patient outcomes, and reduce social burden.

## Materials and methods

### Study design

This study adopted a nested case-control design. First, we identified patients with AIS and AF from 2020 to 2023 as study subjects, collecting inpatient medical records and categorizing them into SAP and non-SAP groups based on in-hospital onset status. Initial screening was performed through univariate analysis of demographic characteristics, clinical indicators, and laboratory parameters. Statistically significant variables were subsequently processed using LASSO regression for dimensionality reduction and final feature selection. The overall sample was then randomly divided into training (70%), validation (20%), and test (10%) sets for model development, parameter optimization, and final evaluation, respectively. Four machine learning models—LR, RF, SVM, and XGBoost—were constructed using the selected features. To address class imbalance in the training set, which could adversely affect predictive performance, we applied a synthetic minority oversampling technique (SMOTE) to achieve balanced class distribution, thereby enhancing prediction accuracy and stability. Model hyperparameters were optimized via cross-validation and grid search on the validation set. Comprehensive evaluation metrics including accuracy, sensitivity, specificity, AUC, Youden index, *F*_1_ score, and decision curve analysis (DCA), were employed on the test set to identify the optimal predictive model. The final model was visualized using nomograms, with decision curve analysis assessing its clinical utility.

In this study, a total of 10,967 patients diagnosed with AIS and AF were identified. Among those, 6,471 were excluded according to the following criteria: 2,341 were under 18 years of age, 2,028 patients had a hospital stay of less than 24 h, 356 had an infection within 1 week before the onset of AIS, 337 had a tumor, and 1,139 had missing values for variable exceeding 20%. Ultimately, 4,496 patients with AIS and AF were enrolled in this study, including 457 cases of SAP during hospitalization, and 4,039 cases did not develop SAP.

### Study population and diagnostic criteria

Inclusion criteria: (1) Meet the diagnostic criteria for AIS as outlined in the Chinese Guidelines for the Diagnosis and Treatment of AIS. (2) Diagnosed with AF either during the current screening electrocardiogram (12-lead electrocardiogram or single-lead electrocardiogram with AF rhythm ≥30 s); or through prior medical diagnoses, electrocardiograms, or medical records. (3) Over 18 of age.

Exclusion criteria: (1) Automatically discharged from hospital within 24 h of admission or died during hospitalization. (2) Lung infection before or shortly before the onset of stroke. (3) With infections affecting other tissues and organs. (4) With cancer, severe liver or kidney dysfunction, severe hematological disorders, or autoimmune diseases. (5) With incomplete medical records.

Diagnostic criteria for SAP refer to the “Chinese Expert Consensus on the Diagnosis and Treatment of SAP” and meet at least one of the following criteria: (1) Fever ≥38°C without identifiable alternative causes. (2) Decreased (≤4 × 10^9^/L) or increased (≥10 × 10^9^/L) peripheral blood leukocyte counts. (3) Elderly persons aged ≥70 years with a sudden change in consciousness. Additionally, patients must fulfill at least 2 of the following secondary criteria: (1) Newly developed cough, increased respiratory rate, or even difficulty breathing. (2) New sputum production or changes in sputum within 24 h. (3) Presence of rales, bronchial breath sounds, or crackling sounds in the lungs. (4) Impaired gas exchange. Chest imaging should demonstrate at least one of the following findings: (1) New or progressive infiltrating shadows. (2) New or progressive solid shading. (3) New or progressive ground-glass shadows. (Note: for patients without prior cardiopulmonary conditions, a single chest imaging examination showing any one of the above manifestations may be sufficient).

### Variables

#### Demographic data

Age, sex, smoking, drinking, and medical history (whether it was the first cerebral infarction, coronary heart disease, hypertension, diabetes, and duration of AF), medication history (anticoagulants, antiplatelet medications, antihypertensive medications, lipid-lowering medications, and heart rate controlling medications), and hospital day.

#### Clinical data

Body temperature, heart rate, respiration rate, diastolic blood pressure (DBP), systolic blood pressure (SBP), admission date, consciousness disorders, cognitive disorders, limb movement disorders, dysphagia, oxygen intake, and nasal feeding requirements.

#### Laboratory data

Red blood cell count (RBC), white blood cell count (WBC), hemoglobin (HB), hematocrit (HCT), platelet count (PLT), platelet crit (PCT), neutrophil count (NEUT), lymphocyte count (LYM), monocyte count (MONO), eosinophil (EO), basophil (BA), neutrophil ratio (NEUT%), C-reactive protein (CPR), international normalized ratio (INR), prothrombin time (PT), activated partial prothrombin time (APTT), D-dimer (DD), fibrinogen (FIB), albumin (ALB), direct bilirubin (DBIL), creatinine (Cr), total cholesterol (TC), homocysteine (HCY), low-density lipoprotein (LDL), and high-density lipoprotein (HDL).

Inflammatory markers calculated using formulae: PLR = platelet count/lymphocyte count, NLR = neutrophil count/lymphocyte count, MLR = monocyte count/lymphocyte count, NRAP = neutrophil percentage/albumin, SIRI = (neutrophil count × monocyte count)/lymphocyte count, SII = platelets count × (neutrophil count/lymphocyte count), CAR = C-reactive protein/albumin.

### Data analysis

Before establishing the model, whether SAP occurred in patients with AIS and AF was evaluated. These patients were divided into two groups: the SAP group and the non-SAP group. Univariate analysis was used to describe the demographic information, clinical data, and laboratory indicators of AIS and AF patients. The statistically significant variables identified from univariate analyses were then included in the LASSO regression to determine the predictors for inclusion in the model. When building the model, AIS, and AF patients were randomly divided into three sets: the training set, validation set, and test set, according to the ratio of 7:2:1. Due to the significant imbalance between the positive and negative samples, a SMOTE was utilized to balance the datasets within the training set.

The predictors were included in the training set after SMOTE balance, which was used to construct LR, RF, SVM, and XGBoost models. The validation set was employed to adjust model parameters and optimize model performance. In the LR, a stepwise regression approach was utilized for multifactor logistic regression analysis. The RF model utilized bootstrap sampling along with five-fold cross-validation. For the SVM model, a grid search method combined with 10-fold cross-validation was applied. The XGBoost model incorporated hyperparameter optimization along with 10-fold cross-validation to enhance its performance.

The test set was used to evaluate the performance of four models. We compared the receiver-operating-characteristic (ROC) curves among the models and computed various metrics, including accuracy, sensitivity, specificity, Youden index, *F*_1_ score, and AUC. Construct DCA in the test set. This analysis assisted in identifying the best predictive model, forest map, and nomogram to clarify independent influences on the occurrence of SAP in patients with AF and AIS. Calibration curve to evaluate the performance of a nomogram.

### Statistical analysis

This study described the characteristics of various datasets and performed different statistical tests. For continuous data, we utilized means and standard deviations, or medians and quartiles, to describe the variables. The Kruskal–Wallis rank sum test was applied to compare differences among groups. For categorical data, we described the data using rates and absolute numbers, employing the chi-square test to assess differences between groups. A two-sided *p*-value <0.05 was considered statistically significant. The predictive ability of models was determined based on the AUC value, with the best cutoff point defined as the one that maximized the Youden index. Statistical analyses and model construction were performed using IBM SPSS Statistics (version 26.0) and R (version 4.3.2).

### Ethics approval and consent to participate

Ethics approval was approved by the Medical Ethics Committee of Shandong Provincial Center for Disease Control and Prevention. Because the data are anonymized, the Medical Ethics Committee of Shandong Provincial Center for Disease Control and Prevention agreed to waive informed consent. All research was conducted following national guidelines and regulations.

## Results

### Baseline characteristics

A total of 4,496 patients with AIS and AF were included in this study. The average age was 73.56 ± 9.95 years (mean ± standard), with 2,679 (59.59%) patients being male. Among the total sample, 457 (10.16%) patients developed SAP. The baseline characteristics of the SAP patients are shown in Table 1. Patients in the SAP group were older, higher prevalence of coronary heart disease, hypertension, and diabetes. They also experienced longer hospital stays and exhibited more frequent consciousness disorders, cognitive impairments, limb movement disorders, dysphagia, nasal feeding requirements, and oxygen intake. Additionally, the SAP group showed significantly elevated levels of body temperature, heart rate, respiration rate, systolic blood pressure, diastolic blood pressure, neutrophil count, neutrophil ratio, NLR, NPAR, SIRI, and SII. In contrast, the levels of hematocrit were significantly lower in the SAP group compared to the non-SAP group (*p* < 0.05).

**Table 1:** Baseline characteristics of patients with AIS and AF in the two groups.

Variables	SAP (*n* = 4,039)	Non-SAP (*n* = 457)	*t*/*x^2^*/*Z*	*p*
Demographics				
Age (years, $\bar{x}\pm s$ )	73.4 ± 10	75.1 ± 9.7	−3.51	<0.001
Sex, *n* (%)			1.72	0.181
Female	2,420 (59.9)	259 (56.7)		
Male	1,619 (40.1)	198 (43.3)		
Smoking, *n* (%)	670 (16.6)	76 (16.6)	0.001	0.982
Drinking, *n* (%)	730 (18.1)	91 (19.9)	0.930	0.335
Cerebral infarction, *n* (%)	1,151 (28.5)	150 (32.8)	3.76	0.053
Coronary heart disease, *n* (%)	814 (20.2)	160 (35)	53.4	<0.001
Diabetes, *n* (%)	460 (11.4)	98 (21.4)	38.19	<0.001
Hypertension, *n* (%)	1,235 (30.6)	214 (46.8)	49.63	<0.001
Duration of AF (years, $\bar{x}\pm s$ )	7.38 ± 4.17	7.76 ± 4.86	−1.80	0.072
Anticoagulants, *n* (%)	180 (4.5)	27 (5.9)	1.65	0.158
Antiplatelet drugs, *n* (%)	207 (5.1)	18 (3.9)	0.98	0.309
Antihypertensive drugs, *n* (%)	140 (3.5)	20 (4.4)	0.74	0.349
Lipid lowering drugs, *n* (%)	151 (3.7)	11 (2.4)	1.73	0.184
Antiarrhythmic drugs, *n* (%)	34 (0.8)	7 (1.5)	1.47	0.185
Clinical data				
Hospital day (days, $\bar{x}\pm s$ )	9.09 ± 5.30	11.07 ± 6.44	−6.80	<0.001
Consciousness disorder, *n* (%)	263 (6.5)	209 (45.7)	672.15	<0.001
Cognitive disorder, *n* (%)	748 (18.5)	318 (69.6)	591.85	<0.001
Limb movement disorders, *n* (%)	1,013 (25.1)	335 (73.3)	454.79	<0.001
Dysphagia, *n* (%)	67 (1.5)	69 (15.1)	252.79	<0.001
Nasal feeding, *n* (%)	1,770 (43.8)	105 (23)	241.44	<0.001
Oxygen uptake, *n* (%)	1,280 (31.7)	121 (26.5)	644.77	<0.001
Temperature (°C, $\bar{x}\pm s$ )	36.37 ± 0.31	36.46 ± 0.43	−4.17	<0.001
Respiration rate (times/min, $\bar{x}\pm s$ )	18.83 ± 1.83	19.24 ± 2.71	−3.09	<0.002
Heart rate (times/min, $\bar{x}\pm s$ )	88.60 ± 17.87	91.64 ± 20.23	−3.12	<0.002
SBP (mmHg, $\bar{x}\pm s$ )	141.02 ± 20.04	146.35 ± 24.43	−4.50	<0.001
DBP (mmHg, $\bar{x}\pm s$ )	84.16 ± 12.20	86.20 ± 15.17	−2.78	<0.006
Admission date (h, $\bar{x}\pm s$ )	7.85 ± 6.27	7.96 ± 6.47	−0.35	0.725
Laboratory data				
RBC (10^12^/L)	4.51 ± 1.22	4.47 ± 1.18	0.73	0.465
WBC (10^9^/L)	7.41 ± 3.56	7.64 ± 3.69	−1.33	0.184
NEUT (10^9^/L)	6.06 (4.41, 7.43)	6.55 (4.65, 8.00)	−3.97	<0.001
LYM (10^9^/L)	1.98 ± 1.48	2.13 ± 1.77	−1.74	0.082
MONO (10^9^/L)	0.54 (0.38, 0.70)	0.54 (0.36, 0.71)	−0.29	0.769
EO (10^9^/L)	0.23 ± 0.29	0.22 ± 0.29	0.59	0.554
BA (10^9^/L)	0.45 ± 0.33	0.48 ± 0.34	−1.72	0.085
HB (g/L)	130.75 ± 18.79	129.68 ± 19.92	1.149	0.251
NEUT% (%)	64.75 ± 8.05	65.58 ± 6.55	−2.48	0.013
PLT (10^9^/L)	194 (157, 232)	194 (158, 233)	−0.06	0.953
PCT (%)	0.21 ± 0.06	0.21 ± 0.07	−1.23	0.218
HCT (L/L)	0.40 ± 0.70	0.39 ± 0.07	2.22	0.026
CPR (mg/L)	5.58 ± 3.25	5.62 ± 3.29	−0.26	0.796
INR	1.14 ± 0.19	1.14 ± 0.17	0.75	0.452
PT (s)	12.91 ± 1.48	12.97 ± 1.45	−0.74	0.457
APTT (s)	31.70 ± 4.92	31.96 ± 4.92	−1.09	0.276
DD (mg/L)	0.36 ± 0.21	0.36 ± 0.22	0.33	0.735
FIB (g/L)	3.24 ± 0.81	3.28 ± 0.83	−1.08	0.280
ALB (g/L)	37.42 ± 4.86	37.05 ± 5.25	1.47	0.143
DBIL (μmol/L)	4.78 ± 2.23	4.79 ± 2.23	−0.44	0.965
Cr (mg/dL)	76.74 ± 25.68	75.84 ± 27.37	0.71	0.480
TC (mmol/L)	4.02 ± 1.09	3.97 ± 1.04	0.88	0.377
LDL (mmol/L)	2.42 ± 0.82	2.39 ± 0.81	0.66	0.509
HDL (mmol/L)	1.21 ± 0.27	1.21 ± 0.28	0.34	0.731
HCY (μmol/L)	13.20 ± 3.28	13.18 ± 3.49	0.12	0.906
PLR	125.00 (89.22, 171.81)	129.41 (79.22, 181.66)	−0.68	0.497
NLR	4.13 ± 2.52	4.49 ± 2.91	−2.55	0.011
MLR	0.36 ± 0.23	0.38 ± 0.25	−1.23	0.220
NPAR	1.73 (1.55, 1.95)	1.80 (1.62, 2.00)	−3.76	<0.001
SIRI	2.30 ± 1.97	2.56 ± 2.28	−2.36	0.019
SII	822.53 ± 587.33	899.73 ± 678.67	−2.34	0.020
CAP	0.15 ± 0.09	0.16 ± 0.10	−0.88	0.401

### Predictors screened by LASSO regression analysis

**Figure 1:**
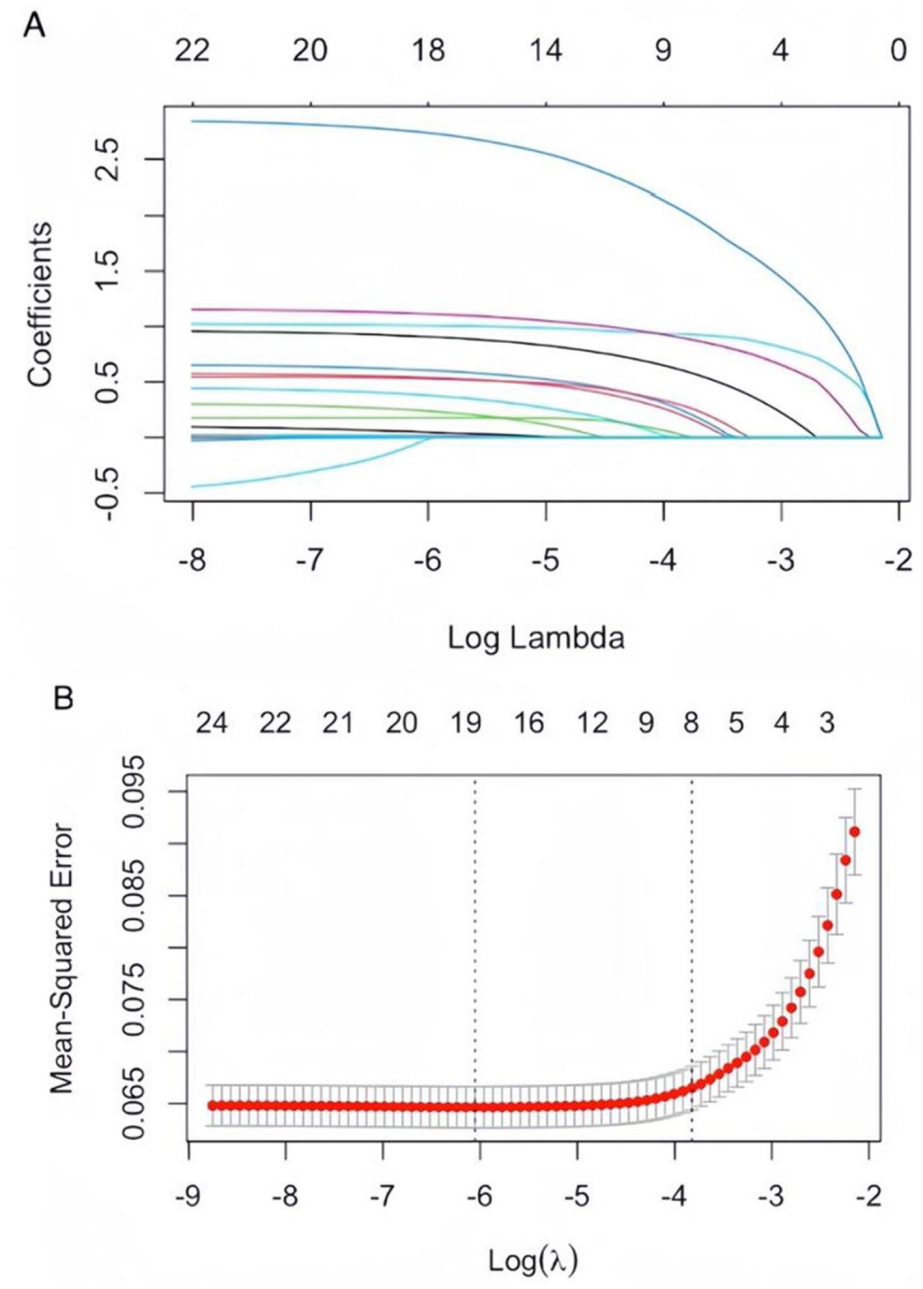
**(A)** LASSO coefficient path plot: as the lambda value increases, the coefficients of the features are compressed toward zero. When a coefficient is compressed to zero, it means that the corresponding variable has been eliminated from the model. The later a variable’s coefficient is compressed to zero, the more influential it is in the model. **(B)** Cross-validation curves for LASSO regression: the solid curve represents the mean cross-validated error, and the region between the two dotted lines indicates the range of positive and negative standard deviations of log (*λ*). The left dotted line corresponds to the value of log (*λ*) at which the model error is minimized. Eight variables were selected when log (*λ*) = −3.8724.

Using SAP as the dependent variable, we included 24 statistically significant variables (*p* < 0.05) identified in prior univariate analysis as independent variables. LASSO regression was employed to screen for predictors, and 10-fold cross-validation was conducted to determine the optimal *λ* value. Ultimately, eight variables were identified as having the best performance with the least number of variables: coronary heart disease, hypertension, consciousness disorder, cognitive impairment, limb movement disorder, dysphagia, nasal feeding requirement, and oxygen intake (Figures 1A,B). These findings highlighted critical risk factors associated with the development of SAP in patients with AIS and AF, aiding in clinical decision-making and targeted interventions.

### Model construction, optimization, and evaluation

AIS and AF patients (*n* = 4,496) were randomly divided into three sets: the training set (*n* = 3,147), validation set (*n* = 903), and test set (*n* = 446), according to the ratio of 7:2:1. In the training set, there were 312 positive samples (SAP) and 2,835 negative samples (non-SAP). We utilized SMOTE to balance the datasets within the training set, and construct LR, RF, SVM, and XGBoost models. The SMOTE is strictly confined to the training set, while both the validation and test sets consistently maintain their original imbalanced distributions. All performance metrics are evaluated exclusively on the non-resampled validation and test sets.

In the validation set optimization model, the results showed that all models exhibited robust performance (AUC > 0.8). The LR model demonstrated the highest discriminative ability (AUC = 0.891), followed by XGBoost (AUC = 0.877), random forest (AUC = 0.868), and SVM (AUC = 0.847). The accuracy and sensitivity of the LR model were 91.36 and 88.42, respectively. The specificity of the RF model was 88.42, and the Youden index of the SVM model was 0.68. LR achieved the highest *F*_1_-score (0.84), outperforming XGBoost (0.83), SVM (0.82), and RF (0.79).

**Figure 2:**
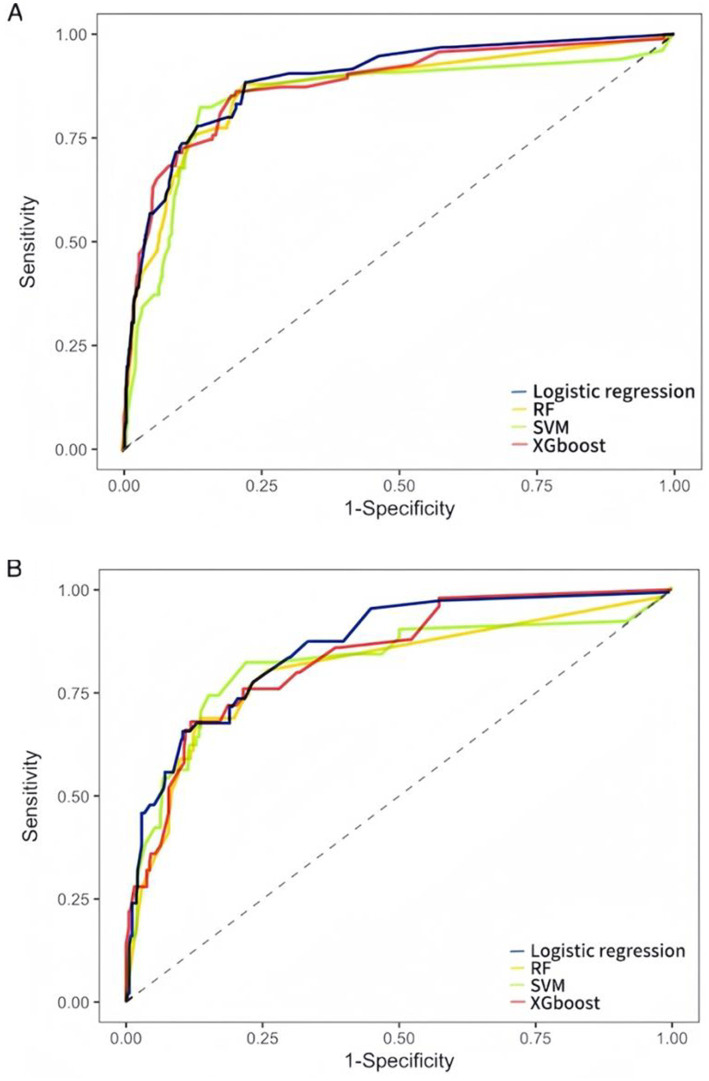
ROC curves of LR, RF, SVM, and XGBoost models for predicting SAP occurrence. **(A)** The validation set ROC curves. **(B)** The test set ROC curves. AUC represents the area under the ROC curve. An AUC value closer to 1 indicates better model performance, while a value closer to 0 indicates poorer performance.

**Figure 3:**
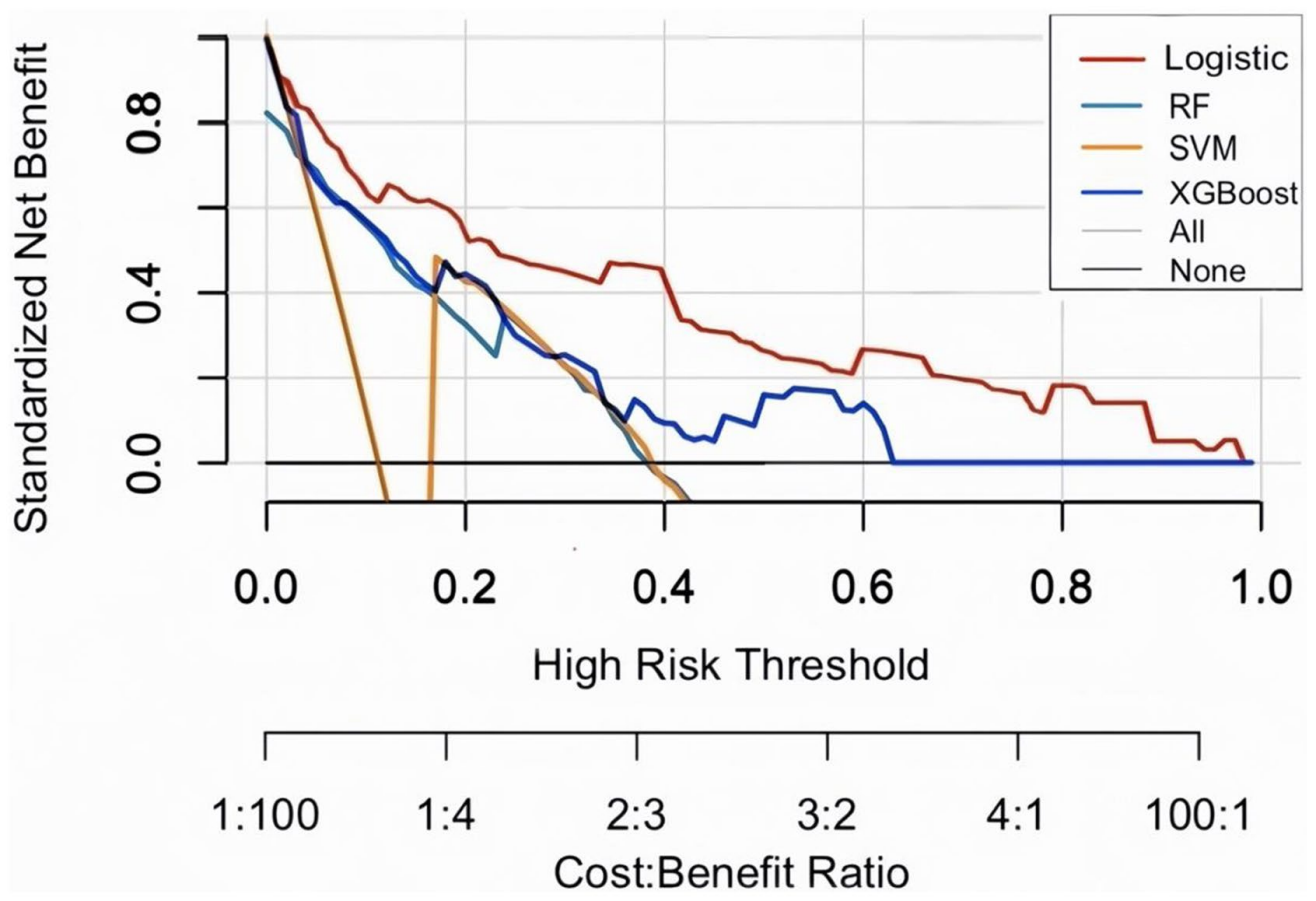
Decision curve analysis (DCA) on test set. The plot displays standardized net benefit (y-axis) against high-risk probability thresholds (x-axis). Higher curves indicate better clinical utility.

Evaluate the model in test set, the AUC (95% CI) for LR, RF, and SVM, XGBoost models were 0.866 (0.8160–917), 0.818 (0.728–0.877), 0.817 (0.780–0.820), and 0.838 (0.780–0.896), respectively (Figures 2A,B). LR had the best predictive performance (AUC = 0.866), highest accuracy (90.13%), sensitivity (79.49%), and Youden index (0.63) in models. LR maintained its lead (0.80) with SVM following closely (0.78), while both XGBoost (0.71) and RF (0.72) showed significant performance drops (Table 2). The DCA showed that the LR model achieved superior net benefit over a broad threshold probability range compared to RF, SVM, and XGBoost models (Figure 3). Among these, the LR model demonstrated the optimal performance in both the validation set and the test set.

**Table 2:** Prediction performance of four models for SAP in patients with AIS and AF.

Variables	Accuracy (%)	Sensitivity (%)	Specificity (%)	AUC (95% CI)	Youden index	*F*_1_ score
Validation set						
LR	91.36	88.42	77.97	0.891 (0.855–0.927)	0.66	0.84
RF	87.49	78.09	88.42	0.868 (0.826–0.906)	0.67	0.79
SVM	87.04	82.10	86.39	0.847 (0.821–0.864)	0.68	0.82
XGBoost	87.26	86.32	79.33	0.877 (0.836–0.918)	0.66	0.83
Test set						
LR	90.13	79.49	83.04	0.866 (0.816–0.917)	0.63	0.80
RF	84.98	70.00	86.11	0.818 (0.728–0.877)	0.56	0.72
SVM	84.30	78.03	82.03	0.817 (0.780–0.820)	0.60	0.78
XGBoost	84.61	66.01	88.64	0.838 (0.780–0.896)	0.55	0.71

### Optimal model and independent influences

**Figure 4:**
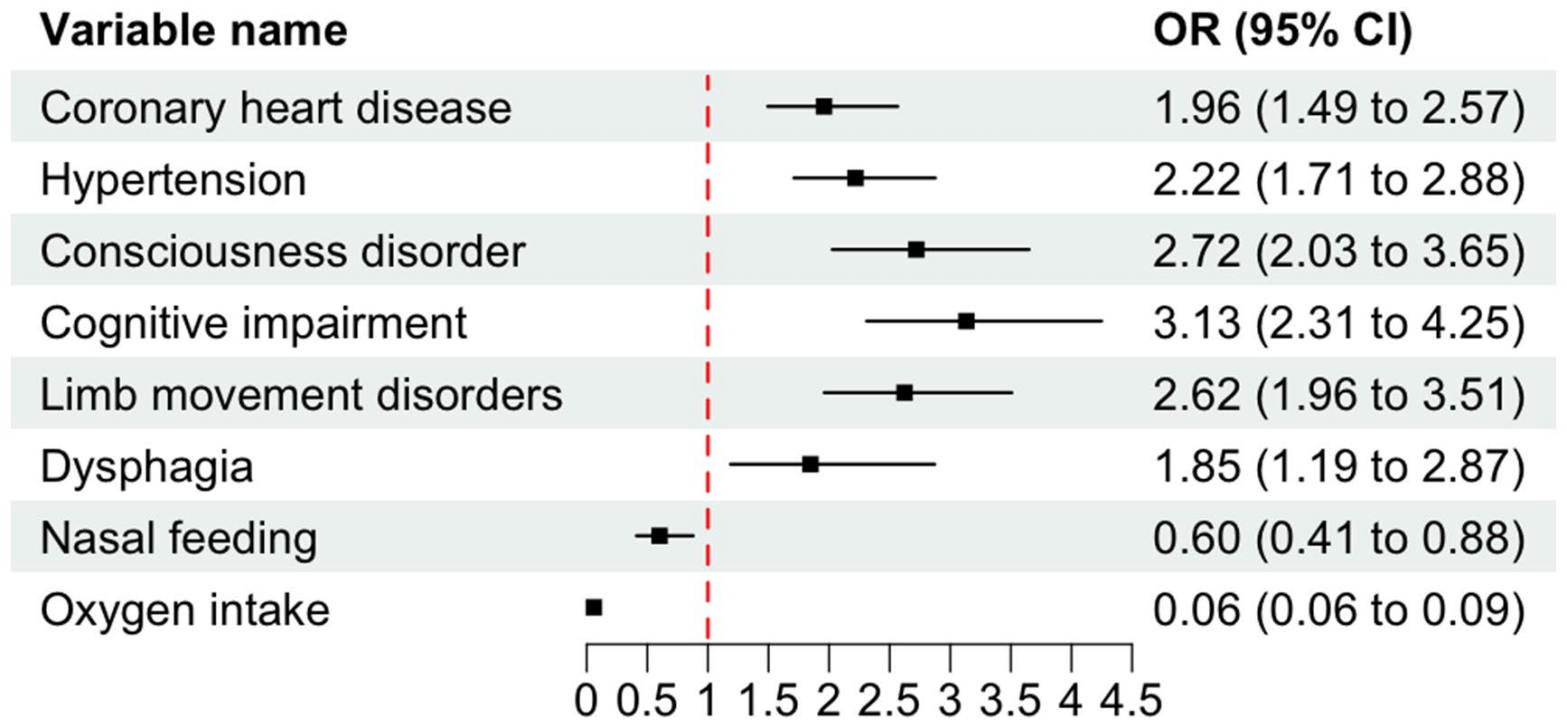
Forest plot of odds ratios (OR) with 95% confidence intervals (CI) for variables associated with SAP. The dashed vertical line indicates the null effect (OR = 1). Error bars represent 95% CIs intervals not crossing the null line denote statistical significance (*p* < 0.05). OR < 1 indicates protective effects, while OR > 1 indicates increased risk.

The findings from the LR model indicated that coronary heart disease, hypertension, consciousness disorder, cognitive impairment, limb movement disorders, dysphagia, nasal feeding, and oxygen intake were independent factors influencing the occurrence of SAP in patients with AIS and AF (*p* < 0.05). Specifically, coronary heart disease [OR = 1.958, 95% CI (1.494–2.566), *p* < 0.001], hypertension [OR = 2.218, 95% CI (1.710–2.876), *p* < 0.001], consciousness disorder [OR = 2.720, 95% CI (2.027–3.650), *p* < 0.001], cognitive impairment [OR = 3.133, 95% CI (2.310–4.248), *p* < 0.001], limb movement disorders [OR = 2.623, 95% CI (1.961–3.507), *p* < 0.001], and dysphagia [OR = 1.846, 95% CI (1.187–2.871), *p* = 0.006] were identified as risk factors for SAP in patients with AIS and AF (OR > 1). In contrast, nasal feeding [OR = 0.601, 95% CI (0.411–0.878), *p* = 0.008], and oxygen intake [OR = 0.059, 95% CI (0.040–0.087), *p* < 0.001] were recognized as protective factors (OR < 1) (Table 3 and Figure 4).

**Table 3:** LR analysis results.

Variables	*B*	SE	Wald*χ*^2^	OR (95% CI)	*p*
Coronary heart disease	0.6681	0.1378	23.712	1.958 (1.494, 2.566)	<0.001
Hypertension	0.7941	0.1324	36.055	2.218 (1.710, 2.876)	<0.001
Consciousness disorder	1.0186	0.1504	44.503	2.720 (2.027, 3.650)	<0.001
Cognitive impairment	1.1442	0.1551	54.028	3.133 (2.310, 4.248)	<0.001
Limb movement disorders	0.9721	0.1482	42.248	2.623 (1.961, 3.507)	<0.001
Dysphagia	0.6324	0.2260	7.415	1.846 (1.187, 2.871)	0.006
Nasal feeding	−0.5097	0.1933	6.039	0.601 (0.411, 0.878)	0.008
Oxygen intake	−2.8299	0.1981	204.142	0.059 (0.040, 0.087)	<0.001

**Figure 5:**
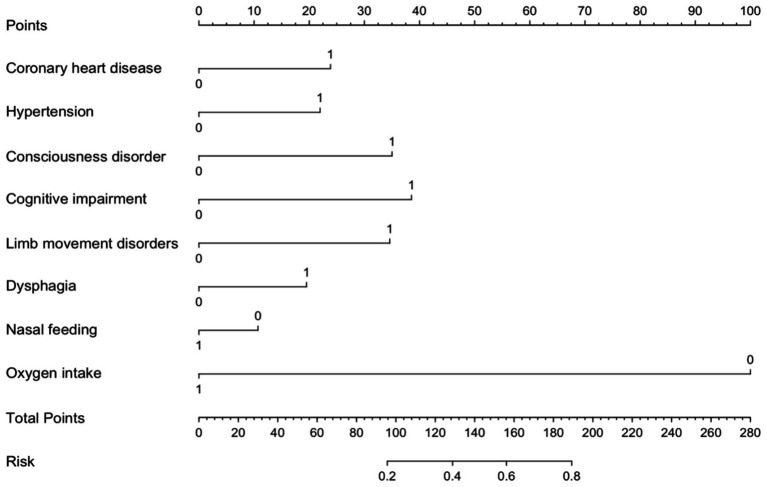
Nomogram for predicting SAP risk in AIS patients with AF. The nomogram integrates multiple predictors to estimate the risk of SAP in AIS patients AF. Each predictor is assigned a score on the point scale, and the total points correspond to the predicted probability of SAP. Higher total points indicate a greater risk of SAP.

**Figure 6:**
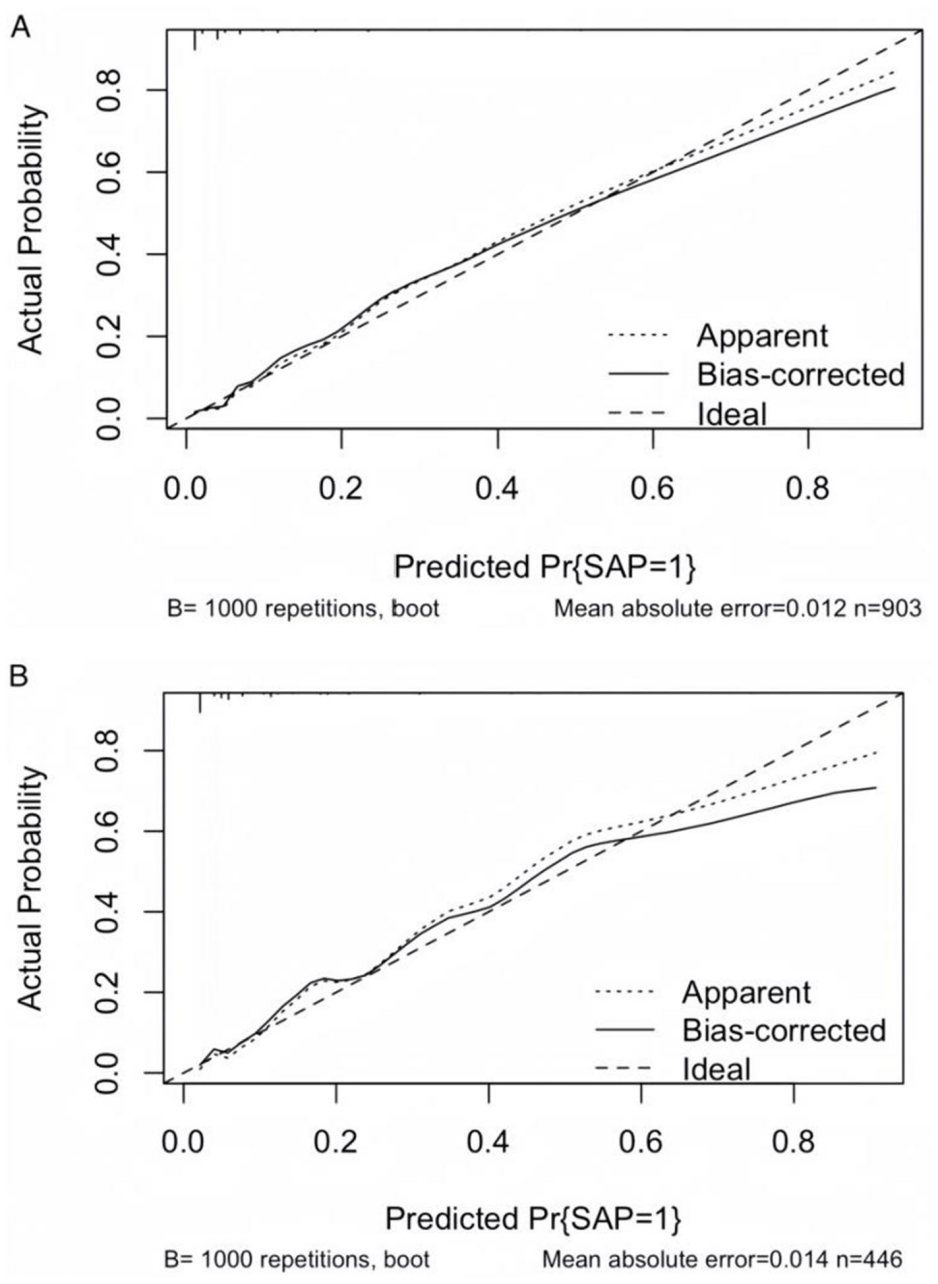
Calibration curves of the LR model. **(A)** Calibration curve of the LR model in the validation set. **(B)** Calibration curve of the LR model in the test set. The diagonal dashed line represents perfect calibration, where predicted probabilities match observed probabilities. Points closer to the diagonal indicate better model calibration.

To facilitate the clinical service, we converted the complex mathematical model into a nomogram (Figure 5). It was necessary to sum the scores of variables included in the model. And then a vertical line at the total score was drawn and making it intersect with the one line representing the predicted SAP. The calibration curve demonstrated that the LR model predicted the risk of SAP in patients with AIS and AF with good consistency to the actual risk, as indicated by the curve fitting closely to the diagonal of the calibration chart, which reflects the agreement between observed results and predicted probabilities. The mean absolute error in the validation set and the test set were 0.012 and 0.014, respectively (Figures 6A,B). It shows that the nomogram had good distinguishing ability.

## Discussion

AIS pathophysiological mechanisms are closely related to immunity. It can disrupt the balance between immunity and the central nervous system by activating the autonomic nervous system and the stress axis, which leads to secondary immune deficiency and increases the risk of infections and SAP (Meisel et al., 2005). After AIS, the inflammatory reaction, as a defense mechanism against infection, promotes tissue regeneration and removal of necrotic cells. However, an excessive inflammatory response can lead to secondary injury. Pneumonia is the most common type of infection following AIS and significantly impacts the recovery of neurological function (Hotter et al., 2020).

Immunoinflammatory markers, including neutrophil-lymphocyte ratio (NLR), platelet-lymphocyte ratio (PLR), and systemic immune-inflammatory index (SII)—a composite of neutrophil, platelet, and lymphocyte counts—are widely used to assess inflammatory responses in malignancies and infections, while prior studies highlight their predictive value for pneumonia (Liu et al., 2018). SIRI, SII, and NLR were more predictive of pneumonia than traditional inflammatory factors (Chang et al., 2021). In this study, SII and SIRI were introduced as markers to assess the overall immune and inflammatory status of SAP in patients with AIS and AF. However, our findings revealed reduced predictive utility of these indices in AF-associated AIS patients. This discrepancy may stem from the incomplete understanding of the pathogenesis of AF, where atrial electrical remodeling serves as a crucial pathophysiological mechanism (Wang et al., 2023). Patients with AF typically exhibit weakened immune function and are prone to inducing inflammation. At the same time, inflammation may promote the development of AF, thus creating a vicious circle between the two (Korantzopoulos et al., 2018). Numerous studies have confirmed the various immune inflammatory markers, such as CPR, interleukins, white blood cell count, are significantly higher in patients with AF compared to those without AF (Ihara and Sasano, 2022). Elevated inflammatory markers in AF patients may result from pre-existing conditions, which increase systemic inflammation even prior to AIS onset. Consequently, future research should include longitudinal immune inflammatory markers to improve the accuracy of prediction.

This study identified a 10.16% incidence of SAP in patients with AIS and AF, lower than rates in severe stroke populations but higher than general hospitalized patients. Key risk factors included hypertension, diabetes, consciousness disorder, dysphagia, cognitive impairment, and limb movement disorders all significantly more prevalent in the SAP group, impaired physical function in elderly patients combined with other chronic diseases may also lead to SAP. In this study along with previous findings and are acknowledged to increase the risk of SAP (Nso et al., 2020; Chang et al., 2022). These comorbidities can contribute to a more complex clinical picture, making patients more vulnerable to respiratory complications.

Nasal feeding remains a controversial topic. It is typically used in patients with dysphagia to provide essential nutritional support, helping to mitigate the risk of malnutrition and related complications (Tinker et al., 2021). In clinical practice, enteral nutrition is generally considered as the first choice, and feeding via nasogastric tube is more common in Asian countries (Galovic et al., 2019). Studies have confirmed that nasal feeding in patients with massive cerebral infarction could effectively correct metabolic disorders, promote neurological recovery, and reduce the occurrence of related infections (Minelli et al., 2022). The findings of our study align with previous study, indicating that nasal feeding may act as a protective factor against SAP in patients with AIS and AF, thereby reducing the incidence of this complication. However, prolonged use of nasogastric feeding may increase the risk of nasal infection, thereby raising the likelihood of SAP in turn (Patel et al., 2020). Therefore, it is crucial to maintain cleanliness of the nasal passages and to regularly change the nasal tubes to minimize the chance of infection when implementing nasal feeding therapy.

In this study, oxygen intake was identified as a protective factor for SAP in patients with AIS and AF. Since its introduction in 1855, supplemental oxygen has been widely used in acute care, and physicians consider it a harmless and potentially beneficial treatments, even in the absence of hypoxemia (Park et al., 2021). Our research results also demonstrated the same conclusion. However, a systematic review and meta-analysis of more than 16,000 patients with acute illnesses have indicated that supplemental oxygen levels exceeding the range of SpO_2_ (94–96%) may lead to vasoconstriction in the pulmonary, cardiovascular, and neurological systems, as well as inflammatory responses, and oxidative stress, potentially resulting in life-threatening conditions (Shultz and Hartmann, 2005). Therefore, when administering oxygen to improve clinical symptoms in patients with AIS, it is crucial for clinicians to conduct thorough assessment before providing oxygen, to minimize the risk of pulmonary inflammation.

The data for this study were extracted from the web-based reporting system of 24 hospitals in Shandong Province, China. Given potential variations in assessment criteria across hospitals of different tiers, NIHSS and CHA₂DS₂-VASc scores were excluded from our analysis to minimize potential bias. The NIHSS scores is a widely used tool for assessing neurological deficits in stroke patients, with higher scores indicating more severe neurological impairment. Studies have demonstrated a linear correlation between NIHSS scores and patient prognosis, where elevated admission scores are associated with increased risks of adverse outcomes (Cheng et al., 2022). However, certain NIHSS components (including facial palsy, ataxia, and gaze assessment) showed significant scoring variations due to subjective interpretation differences (Kasner, 2006). Since standardized assessment protocols were not implemented across study sites during initial data collection, we observed substantial scoring inconsistencies, there are excessive outliers and extremes. Consequently, NIHSS scores were excluded from our final analysis to ensure data reliability. The CHA₂DS₂-VASc score, while validated for stroke risk assessment in atrial fibrillation patients (Requena Calleja et al., 2019), was excluded from our analysis due to incomplete data (>50% missing components). This composite metric incorporates congestive heart failure, prior stroke and vascular disease, but could not be reliably calculated in our cohort. We therefore could not assess its prognostic value for cognitive outcomes in this population.

Based on a large-scale cohort of 4,496 patients with AIS and AF, this study systematically evaluated the performance of LR, RF, SVM, and XGBoost machine learning models in predicting SAP. The results demonstrated that the LR model achieved superior overall performance (AUC = 0.866) and calibration consistency compared to complex ensemble algorithms, outperforming RF, SVM, and XGBoost in predicting SAP. Additionally, the LR model demonstrated superior clinical utility in predicting SAP after intracerebral hemorrhage, outperforming RF, SVM, and XGBoost models (Chu et al., 2018). Although prior studies suggest XGBoost may excel in specific scenarios (Zhang et al., 2024). In our study confirms that LR remains the gold standard for balancing predictive accuracy and clinical utility in large-scale datasets dominated by linear associations. Compared with complex ensemble algorithms, the predictive advantage of LR model in SAP may be because the linear feature screening mechanism is more suitable for clinical data and provides a transparent decision-making framework for clinical practice while mitigating interference from high-dimensional noise (Greenland et al., 2016). In clinical applications, the choice of predictive models is intrinsically linked to dataset scale. For small-to-medium-sized datasets (*n* < 1,000), penalized linear models such as LR generally outperform complex machine learning methods due to their superior interpretability and robustness (Steyerberg, 2019). A meta-analysis of 112 clinical prediction modeling studies demonstrated that the difference in AUC between LR and sophisticated algorithms was consistently <0.02. The inherent simplicity and transparency of LR models facilitate both clinical interpretation and practical implementation. Notably, linear feature selection methods based on LR demonstrate exceptional compatibility with clinical data, exhibiting marked advantages in disease assessment and diagnostic differentiation compared to alternative approaches (Christodoulou et al., 2019). Future research should explore multimodal integration of LR with radiomics or dynamic biomarker monitoring to enhance real-time predictive capabilities.

This study possesses several limitations. Firstly, due to the varying levels of hospital comprehensiveness and differing judgmental criteria among clinicians, CHA₂DS₂-VASc scores, and NIHSS scores were not included, which limited the ability to assess the severity of the patients’ conditions. Secondly, while the target population comprised patients with AIS and AF, it remains unverified whether the model can be generalized to the border population, highlighting the need to consider its applicability across different demographics. Lastly, this study was validated and tested hospitals in Shandong Province, and further validation is required to determine its relevance in other regions.

## Conclusion

In this study, we used a large-sample, multi-center case–control design to identify the risk factors for SAP in patients with AIS and AF. We systematically analyzed coronary artery disease, hypertension, consciousness disorder, cognitive impairment, limb movement disorder, dysphagia, nasal feeding, and oxygen intake as independent influencing factor of SAP. Through comparative evaluation of four machine learning algorithms—LR, RF, SVM, and XGBoost—the risk prediction model based on LR demonstrated the optimal predictive efficacy for SAP in patients with AIS complicated by AF. The derived nomogram serves as a clinically interpretable visualization tool, providing actionable evidence to guide risk stratification and individualized intervention strategies in future clinical research and practice.

## Data Availability

The datasets used and/or analyzed during the current work are available from the corresponding author on reasonable request.

## Glossary

SAP: Stroke-associated pneumonia
AIS: Acute ischemic stroke
AF: Atrial fibrillation
LASSO: Least absolute shrinkage and selection operator
SMOTE: Synthetic minority oversampling technique
RF: Random forest
SVM: Support vector machine
XGBoost: Extreme gradient boosting
DBP: Diastolic blood pressure
SBP: Systolic blood pressure
RBC: Red blood cell count
WBC: White blood cell count
HB: Hemoglobin
HCT: Hematocrit
PLT: Platelet count
PCT: Plateletcrit
NEUT: Neutrophil count
LYM: Lymphocyte count
MONO: Monocyte count
EO: Eosinophil
BA: Basophil
NEUT%: Neutrophil ratio
CPR: C-reactive protein
INR: International normalized ratio
PT: Prothrombin time
APTT: Activated partial prothrombin time
DD: D-dimer
FIB: Fibrinogen
ALB: Albumin
DBIL: Direct bilirubin
Cr: Creatinine
TC: Total cholesterol
HCY: Homocysteine
LDL: Low-density lipoprotein
HDL: High-density lipoprotein
ROC: Receiver-operating-characteristic
AUC: Area under the receiver operating characteristic curve
CI: Confidence interval

## References

[ref1] Bai Q. Liu J. Wang G. (2020). Ferroptosis, a regulated neuronal cell death type after intracerebral hemorrhage. Front. Cell. Neurosci. 14:591874. doi: 10.3389/fncel.2020.591874, PMID: 33304242 PMC7701249

[ref2] Cerami C. Perani D. (2015). Imaging neuroinflammation in ischemic stroke and in the atherosclerotic vascular disease. Curr. Vasc. Pharmacol. 13, 218–222. doi: 10.2174/15701611113116660168, PMID: 24188483

[ref3] Chang M. C. Choo Y. J. Seo K. C. Yang S. (2022). The relationship between dysphagia and pneumonia in acute stroke patients: a systematic review and meta-analysis. Front. Neurol. 13:834240. doi: 10.3389/fneur.2022.834240, PMID: 35370927 PMC8970315

[ref4] Chang T. I. Wu X. Boström K. I. Tran H. A. Friedlander A. H. (2021). Red cell distribution width, unlike neutrophil lymphocyte ratio is unable to accurately gauge enhanced systemic inflammation associated with panoramic imaged carotid plaque. Mil. Med. 186, 39–43. doi: 10.1093/milmed/usaa302, PMID: 33005942 PMC12915552

[ref5] Cheng X. Q. Shi J. Q. Wu H. Dong Z. Liu J. Zhou C. S.. (2022). ASPECTS-based attenuation changes on CT angiography as an imaging biomarker to predict hemorrhagic transformation in acute ischemic stroke. Cerebrovasc. Dis. 51, 532–541. doi: 10.1159/000521026, PMID: 34983042

[ref6] Christodoulou E. Ma J. Collins G. S. Steyerberg E. W. Verbakel J. Y. Van Calster B. (2019). A systematic review shows no performance benefit of machine learning over logistic regression for clinical prediction models. J. Clin. Epidemiol. 110, 12–22. doi: 10.1016/j.jclinepi.2019.02.004, PMID: 30763612

[ref7] Chu D. K. Kim L. H. Young P. J. Zamiri N. Almenawer S. A. Jaeschke R.. (2018). Mortality and morbidity in acutely ill adults treated with liberal versus conservative oxygen therapy (IOTA): a systematic review and meta-analysis. Lancet 391, 1693–1705. doi: 10.1016/S0140-6736(18)30479-3, PMID: 29726345

[ref8] Galovic M. Stauber A. J. Leisi N. Krammer W. Brugger F. Vehoff J.. (2019). Development and validation of a prognostic model of swallowing recovery and enteral tube feeding after ischemic stroke. JAMA Neurol. 76, 561–570. doi: 10.1001/jamaneurol.2018.4858, PMID: 30742198 PMC6515605

[ref9] GBD 2019 Stroke Collaborators (2021). Global, regional, and national burden of stroke and its risk factors, 1990–2019: a systematic analysis for the Global Burden of Disease Study 2019. Lancet Neurol. 20, 795–820. doi: 10.1016/S1474-4422(21)00252-0, PMID: 34487721 PMC8443449

[ref10] Gittins M. Lobo Chaves M. A. Vail A. Smith C. J. (2023). Does stroke-associated pneumonia play an important role on risk of in-hospital mortality associated with severe stroke? A four-way decomposition analysis of a national cohort of stroke patients. Int. J. Stroke 18, 1092–1101. doi: 10.1177/17474930231177881, PMID: 37170807 PMC10614175

[ref11] Greenland S. Senn S. J. Rothman K. J. Carlin J. B. Poole C. Goodman S. N.. (2016). Statistical tests, *p* values, confidence intervals, and power: a guide to misinterpretations. Eur. J. Epidemiol. 31, 337–350. doi: 10.1007/s10654-016-0149-3, PMID: 27209009 PMC4877414

[ref12] Hotter B. Hoffmann S. Ulm L. Montaner J. Bustamante A. Meisel C.. (2020). Inflammatory and stress markers predicting pneumonia, outcome, and etiology in patients with stroke: biomarkers for predicting pneumonia, functional outcome, and death after stroke. Neurol. Neuroimmunol. Neuroinflamm. 7:e692. doi: 10.1212/NXI.0000000000000692, PMID: 32098866 PMC7051196

[ref13] Hu J. Szymczak S. (2023). A review on longitudinal data analysis with random forest. Brief. Bioinform. 24:bbad002. doi: 10.1093/bib/bbad002, PMID: 36653905 PMC10025446

[ref14] Ihara K. Sasano T. (2022). Role of inflammation in the pathogenesis of atrial fibrillation. Front. Physiol. 13:862164. doi: 10.3389/fphys.2022.862164, PMID: 35492601 PMC9047861

[ref15] Ji R. Wang D. Shen H. Pan Y. Liu G. Wang P.. (2013). Interrelationship among common medical complications after acute stroke: pneumonia plays an important role. Stroke 44, 3436–3444. doi: 10.1161/STROKEAHA.113.001931, PMID: 24178914

[ref16] Kasner S. E. (2006). Clinical interpretation and use of stroke scales. Lancet Neurol. 5, 603–612. doi: 10.1016/S1474-4422(06)70495-1 16781990

[ref17] Keller K. Hobohm L. Wenzel P. Münzel T. Espinola-Klein C. Ostad M. A.. (2020). Impact of atrial fibrillation/flutter on the in-hospital mortality of ischemic stroke patients. Heart Rhythm. 17, 383–390. doi: 10.1016/j.hrthm.2019.10.001, PMID: 31589988

[ref18] Kelley R. E. Kelley B. P. (2021). Heart-brain relationship in stroke. Biomedicines 9:1835. doi: 10.3390/biomedicines9121835, PMID: 34944651 PMC8698726

[ref19] Kimura T. Kashimura S. Nishiyama T. Katsumata Y. Inagawa K. Ikegami Y.. (2018). Asymptomatic cerebral infarction during catheter ablation for atrial fibrillation: comparing uninterrupted rivaroxaban and warfarin (ASCERTAIN). JACC Clin. Electrophysiol. 4, 1598–1609. doi: 10.1016/j.jacep.2018.08.003 30573125

[ref20] Koennecke H. C. Belz W. Berfelde D. Endres M. Fitzek S. Hamilton F.. (2011). Factors influencing in-hospital mortality and morbidity in patients treated on a stroke unit. Neurology 77, 965–972. doi: 10.1212/WNL.0b013e31822dc795, PMID: 21865573

[ref21] Korantzopoulos P. Letsas K. P. Tse G. Fragakis N. Goudis C. A. Liu T. (2018). Inflammation and atrial fibrillation: a comprehensive review. J. Arrhythm. 34, 394–401. doi: 10.1002/joa3.12077, PMID: 30167010 PMC6111477

[ref22] Li Y. Zou Z. Gao Z. Wang Y. Xiao M. Xu C.. (2022). Prediction of lung cancer risk in Chinese population with genetic-environment factor using extreme gradient boosting. Cancer Med. 11, 4469–4478. doi: 10.1002/cam4.4800, PMID: 35499292 PMC9741969

[ref23] Liu D. D. Chu S. F. Chen C. Yang P. F. Chen N. H. He X. (2018). Research progress in stroke-induced immunodepression syndrome (SIDS) and stroke-associated pneumonia (SAP). Neurochem. Int. 114, 42–54. doi: 10.1016/j.neuint.2018.01.002, PMID: 29317279

[ref24] Meisel C. Schwab J. M. Prass K. Meisel A. Dirnagl U. (2005). Central nervous system injury-induced immune deficiency syndrome. Nat. Rev. Neurosci. 6, 775–786. doi: 10.1038/nrn1765, PMID: 16163382

[ref25] Minelli C. Luvizutto G. J. Cacho R. O. Neves L. O. SCSA M. MTA P.. (2022). Brazilian practice guidelines for stroke rehabilitation: part II. Arq. Neuropsiquiatr. 80, 741–758. doi: 10.1055/s-0042-1757692, PMID: 36254447 PMC9685826

[ref26] Nso N. Bookani K. R. Metzl M. Radparvar F. (2020). Role of inflammation in atrial fibrillation: a comprehensive review of current knowledge. J. Arrhythm. 37, 1–10. doi: 10.1002/joa3.12473, PMID: 33664879 PMC7896450

[ref27] Park J. H. Kim E. Cho H. Park D. W. Choi J. Jang S. H. (2021). Brain activation in response to visual sexual stimuli in male patients with right middle cerebral artery infarction: the first case-control functional magnetic resonance imaging study. Medicine 100:e25823. doi: 10.1097/MD.0000000000025823, PMID: 34032696 PMC8154462

[ref28] Patel U. K. Kodumuri N. Dave M. Lekshminarayanan A. Khan N. Kavi T.. (2020). Stroke-associated pneumonia: a retrospective study of risk factors and outcomes. Neurologist 25, 39–48. doi: 10.1097/NRL.0000000000000269, PMID: 32358460

[ref29] Requena Calleja M. A. Arenas Miquélez A. Díez-Manglano J. Gullón A. Pose A. Formiga F.. (2019). Sarcopenia, frailty, cognitive impairment and mortality in elderly patients with non-valvular atrial fibrillation. Rev. Clin. Esp. 219, 424–432. doi: 10.1016/j.rce.2019.04.001, PMID: 31109685

[ref30] Shultz S. M. Hartmann P. M. (2005). George E Holtzapple (1862–1946) and oxygen therapy for lobar pneumonia: the first reported case (1887) and a review of the contemporary literature to 1899. J. Med. Biogr. 13, 201–206. doi: 10.1177/096777200501300405, PMID: 16244712

[ref31] Shurrab S. Guerra-Manzanares A. Magid A. Piechowski-Jozwiak B. Atashzar S. F. Shamout F. E. (2024). Multimodal machine learning for stroke prognosis and diagnosis: a systematic review. IEEE J. Biomed. Health Inform. 28, 6958–6973. doi: 10.1109/JBHI.2024.3448238 39172620

[ref32] Si Q. Yang Z. Ye J. (2023). Symmetric LINEX loss twin support vector machine for robust classification and its fast iterative algorithm. Neural Netw. 168, 143–160. doi: 10.1016/j.neunet.2023.08.055, PMID: 37748393

[ref33] Steyerberg E. W. (2019). Clinical prediction models: a practical approach to development, validation, and updating. New York, NY: Springer.

[ref34] Teh W. H. Smith C. J. Barlas R. S. Wood A. D. Bettencourt-Silva J. H. Clark A. B.. (2018). Impact of stroke-associated pneumonia on mortality, length of hospitalization, and functional outcome. Acta Neurol. Scand. 138, 293–300. doi: 10.1111/ane.12956, PMID: 29749062

[ref35] Ting Sim J. Z. Fong Q. W. Huang W. Tan C. H. (2023). Machine learning in medicine: what clinicians should know. Singapore Med. J. 64, 91–97. doi: 10.11622/smedj.2021054, PMID: 34005847 PMC10071847

[ref36] Tinker R. J. Smith C. J. Heal C. Bettencourt-Silva J. H. Metcalf A. K. Potter J. F.. (2021). Predictors of mortality and disability in stroke-associated pneumonia. Acta Neurol. Belg. 121, 379–385. doi: 10.1007/s13760-020-01542-9, PMID: 31037709 PMC7956938

[ref37] Wang R. H. Wen W. X. Jiang Z. P. du Z. P. Ma Z. H. Lu A. L.. (2023). The clinical value of neutrophil-to-lymphocyte ratio (NLR), systemic immune-inflammation index (SII), platelet-to-lymphocyte ratio (PLR) and systemic inflammation response index (SIRI) for predicting the occurrence and severity of pneumonia in patients with intracerebral hemorrhage. Front. Immunol. 14:1115031. doi: 10.3389/fimmu.2023.1115031, PMID: 36860868 PMC9969881

[ref38] Xu M. Wang J. Zhan C. Zhou Y. Luo Z. Yang Y.. (2024). Association of follow-up neutrophil-to-lymphocyte ratio and systemic inflammation response index with stroke-associated pneumonia and functional outcomes in cerebral hemorrhage patients: a case-controlled study. Int. J. Surg. 110, 4014–4022. doi: 10.1097/JS9.0000000000001329, PMID: 38498385 PMC11254209

[ref39] Yu Y. J. Weng W. C. Su F. C. Peng T. I. Chien Y. Y. Wu C. L.. (2016). Association between pneumonia in acute stroke stage and 3-year mortality in patients with acute first-ever ischemic stroke. J. Clin. Neurosci. 33, 124–128. doi: 10.1016/j.jocn.2016.02.039, PMID: 27436765

[ref40] Zhang W. Zhou Y. Xu L. Qiu C. Luo Z. Jiang Z.. (2024). Development and validation of radiology-clinical statistical and machine learning model for stroke-associated pneumonia after first intracerebral haemorrhage. BMC Pulm. Med. 24:357. doi: 10.1186/s12890-024-03160-0, PMID: 39048959 PMC11267827

[ref41] Zhao Y. Hua X. Ren X. Ouyang M. Chen C. Li Y.. (2023). Increasing burden of stroke in China: a systematic review and meta-analysis of prevalence, incidence, mortality, and case fatality. Int. J. Stroke 18, 259–267. doi: 10.1177/17474930221135983, PMID: 36274585

